# TGF-β1-mediated exosomal lnc-MMP2-2 increases blood–brain barrier permeability via the miRNA-1207-5p/EPB41L5 axis to promote non-small cell lung cancer brain metastasis

**DOI:** 10.1038/s41419-021-04004-z

**Published:** 2021-07-20

**Authors:** Dongming Wu, Shihua Deng, Li Li, Teng Liu, Ting Zhang, Jing Li, Ye Yu, Ying Xu

**Affiliations:** 1 School of Clinical Medicine, Chengdu Medical College, Chengdu, Sichuan PR China; 2 The First Affiliated Hospital of Chengdu Medical College, Chengdu, Sichuan PR China

**Keywords:** Lung cancer, Metastasis

## Abstract

Brain metastases remain a major problem in patients with advanced non-small cell lung cancer (NSCLC). The permeability of the blood–brain barrier (BBB) is highly increased during lung cancer brain metastasis; however, the underlying mechanism remains largely unknown. We previously found that lnc-MMP2-2 is highly enriched in tumor growth factor (TGF)-β1-mediated exosomes and regulates the migration of lung cancer cells. This study aimed to explore the role of exosomal lnc-MMP2-2 in the regulation of BBB and NSCLC brain metastasis. Here, using endothelial monolayers and mouse models, we found that TGF-β1-mediated NSCLC-derived exosomes efficiently destroyed tight junctions and the integrity of these natural barriers. Overexpression of lnc-MMP2-2 in human brain microvascular endothelial cells increased vascular permeability in endothelial monolayers, whereas inhibition of lnc-MMP2-2 alleviated these effects. Furthermore, lnc-MMP2-2 knockdown markedly reduced NSCLC brain metastasis in vivo. Mechanistically, through luciferase reporter assays, RNA pull-down assay, and Ago2 RNA immunoprecipitation assay, we showed that lnc-MMP2-2 served as a microRNA sponge or a competing endogenous RNA for miR-1207-5p and consequently modulated the derepression of EPB41L5. In conclusion, TGF-β1-mediated exosomal lnc-MMP2-2 increases BBB permeability to promote NSCLC brain metastasis. Thus, exosomal lnc-MMP2-2 may be a potential biomarker and therapeutic target against lung cancer brain metastasis.

## Introduction

Lung cancer is the most common cancer in men and is associated with a high mortality rate worldwide [1]. Distant metastasis remains the primary cause of poor prognosis in lung cancer [2]. Brain metastases are a frequent complication in patients with advanced lung cancer and a major cause of mortality [3]. The blood–brain barrier (BBB) consists of endothelial cells (ECs), lined by pericytes, basement membrane, and astrocytes. This forms a tight barrier around blood vessels to restrict the flow of essential components in and out of the central nervous system (CNS) [4]. The integrity of the BBB is essential in preventing the invasion of tumor cells into the brain. In lung cancer, the BBB permeability is increased in brain metastases [5]. However, the mechanisms associated with the alterations in BBB permeability during lung cancer brain metastases are still unclear.

Exosomes have been recently a topic of interest owing to their role in multiple diseases [6–8]. A previous study has reported that exosomes are early contributors to the pathogenesis of different diseases [9, 10]. Tumor-derived exosomes, which are released from the primary tumor into the circulation, can travel to distant organs and modulate the microenvironment in premetastatic organs to facilitate future metastasis [11]. Tumor growth factor (TGF)-β1 is a multifunctional cytokine, having many biological activities, including regulating cell proliferation, differentiation, and apoptosis. We have previously reported that TGF-β1-mediated non-small cell lung cancer (NSCLC) cell exosomes might increase the permeability of lung vascular ECs and downregulate its tight junctions via lnc-MMP2-2 [12]. However, there is limited data on the significance of exosomal lnc-MMP2-2 in BBB and brain metastasis.

Previous studies have demonstrated that lncRNAs exhibit highly cell type-specific expression and function and have roles in carcinogenesis, metastasis, and drug resistance [13–15]. However, the role of lncRNAs in the regulation of BBB is yet to be fully explored. In this study we explored the role of exosomal lnc-MMP2-2 in the regulation of BBB and NSCLC brain metastasis. Through immunofluorescence (IF) and endothelial permeability assay we determined that lnc-MMP2-2 increases the permeability and destroys the integrity of BBB in vitro and in vivo. To identify the mechanisms by which lnc-MMP2-2 functioned in regulation of BBB permeability, luciferase reporter assays, RNA pull-down, and AGO2-RIP assay were performed and we found that lnc-MMP2-2 served as a microRNA sponge or a competing endogenous RNA (ceRNA) for miR-1207-5p. Furthermore, miR-1207-5p consequently modulated the derepression of EPB41L5. Additionally, we found that lnc-MMP2-2 knockdown drastically reduced NSCLC brain metastasis in mice. We aimed to elucidate the regulatory mechanism of exosomal lnc-MMP2-2 in lung cancer brain pro-metastases and examine whether it could be a promising therapeutic target for patients with NSCLC.

## Materials and methods

### Reagents

Antibodies against N-cadherin (22018-1-AP), ZO-1 (21773-1-AP), occludin (13409-1-AP), CD63 (25682-1-AP), CD81 (27855-1-AP), Alix (12422-1-AP), GAPDH (60004-1-Ig), and anti-Ago2 (10686-1-AP) were purchased from Proteintech (Wuhan, China). Antibodies to claudin-5 (343214) and EPB41L5 (614203) were purchased from Zenbio (Chengdu, China). Anti-VE-cadherin antibodies (2500) were obtained from CST (Beverly, MA, USA). Rhodamine B isothiocyanate-dextran was purchased from Sigma-Aldrich (St. Louis, MO, USA). Exo-Fect Exosome Transfection Kit was purchased from System Biosciences (Beijing, China). Recombinant human transforming growth factor β1 (TGF-β1) was purchased from PeproTech (Rocky Hill, USA).

### Cell cultures and transfections

The NSCLC cell line A549 was obtained from Cell Bank of the Chinese Academy of Sciences (Shanghai, China). Non-fetal-derived human brain microvascular endothelial cells (HBMECs) were purchased from Bioleaf Biotechnology (Shanghai, China). A549 and HBMECs were grown in Roswell Park Memorial Institute-1640 medium (Invitrogen, Carlsbad, CA, USA) containing 10% fetal bovine serum at 37 °C with 5% CO_2_. The lnc-MMP2-2 overexpression and silencing lentivirus and their control lentivirus were packaged by Genomeditech (Shanghai, China). EPB41L5-targeting siRNA and scramble control siRNA were obtained from Ribobio (Guangzhou, China). The EPB41L5-targeting sequences were as follows: siRNA#1, 5′-GGATCACGATTTA GATATA-3′; siRNA#2, 5′-GTCCTGAACTTGTCTCAGA-3′; siRNA#3: 5′-CGACTATTTTGGTCTGAG A-3′. The overexpression plasmid (pcDNA3.1-EPB41L5) and the empty vector (pcDNA3.1) were obtained from Genomeditech (Shanghai, China). miR-1207-5p antagomir, antagomir control, and miR-1207-5p agomir and agomir control were purchased from Ribobio (Guangzhou, China). Cell transfection was performed using Lipofectamine 3000 (Invitrogen, Carlsbad, CA, USA) following the manufacturer’s instructions. For exosome transfection, lnc-MMP2-2 Smart Silencer (RiboBio Co, Ltd., Guangzhou, China) were loaded in exosomes using the Exo-Fect Exosome Transfection Kit.

### Exosome isolation and characterization

Exosomes were isolated according to our previous method [12]. Briefly, exosomes from A549 cells pretreated for 24 h with serum-free media and serum-free media containing 10 ng/ml TGF-β1 were designated as “exo” and “Texo,” respectively. The morphology and particle size of exosomes dissolved in phosphate-buffered saline were characterized via transmission electron microscopy (TEM; FEIG2; FEI, Hillsboro, OR, USA) as previously described.

### Quantitative real-time PCR (qRT-PCR)

qRT-PCR assay was performed as described previously [16]. β-actin or U6 snoRNA was validated as the normalizer. The following primers were used: lnc-MMP2-2-forward, 5′-TCCATCCTGCTGCTCAGTATCTCC-3′ and reverse, 5′-GCTCAGACGTGCCATTCTCAGG-3′. EPB41L5-forward, 5′ATGTTAGTGTGG ACTTGCCAAA-3′ and reverse, 5′-GCAGACAATAGGGTGAACCAAT-3′. β-actin-forward, 5′-CCTGGCACCCAGCACAAT-3′ and reverse, 5′-GGGCCGGACT CG TCATAC-3′.

### Immunofluorescence and western blot

IF and western blotting assays were performed to measure the expressions of VE-cadherin, N-cadherin, occludin, claudin-5, ZO-1, CD63, CD81, Alix, and EPB41L5 according to our previous method [12].

### HBMECs permeability assay

A total of 2000 HBMECs in full medium were seeded on Costar Transwell inserts (0.4-µm pore size; Corning, USA) for 24 h. Rhodamine B isothiocyanate-Dextran (400 µg/ml) was then added to the upper wells. After 2 h of additional incubation at 37 °C, the media in the lower wells were collected, and the fluorescence intensity was measured at excitation and emission wavelengths of 485 and 535 nm, respectively, using a FlexStation 3 microplate reader (USA).

### Detection analysis for BBB integrity in vivo

The BBB integrity was evaluated by using Evans blue (EB) and rhodamine B isothiocyanate-Dextran injection. EB (2% in PBS, 4 ml/kg) or rhodamine B (100 mg/kg) was injected into the mouse tail vein pretreated with exo or Texo for 24 h. After 3 h of circulation, the mice were anesthetized, and the heart was perfused. The mouse brains injected with rhodamine B were embedded into frozen blocks and sectioned into 15 μm by cryostat sectioning. The distribution of rhodamine B in the cerebra was observed through fluorescence microscopy. The EB fluorescence detection assay was performed according to a previous method [17].

### Assembly of lnc-MMP2-2 lentivirus

The lnc-MMP2-2 overexpression, silencing lentivirus, and the empty vector were packaged by Genomeditech (Shanghai, China). The lnc-MMP2-2-silencing targeting sequences were as follows: shControl (NC), 5′-TTCTCCGAACGTGTCACGT-3′; shRNA#1, 5′-GCTGCAAGAAACATCTCTTGC-3′; shRNA#2, 5′-GGATGATCT AGTTCTCCATCC-3′; shRNA#3: 5′-GCCTCCTGC AAATCTCCCAAT-3′. Cell transduction was performed following the manufacturer’s instructions. Stable cells were selected using medium containing 0.5 µg/ml puromycin.

### RNA fluorescence in situ hybridization and luciferase reporter assay

Fluorescence in situ hybridization (FISH) assay was performed according to a previous method [12]. The probe used for lnc-MMP2-2 was 5′-FAM-accctaggctgcaggctcctgctttgggct-3′.

For the luciferase reporter assay, we used the pGL3 luciferase reporter plasmids for lnc-MMP2-2 and EPB41L5 designed by Genomeditech (Shanghai, China). Two reporter plasmids or vector control and control agomir or miR-1207-5p agomir were co-transfected into HBMECs. The luciferase activity was measured as per our method previously described [12].

### RNA immunoprecipitation

RNA immunoprecipitation (RIP) was performed using the EZMagna RIP kit (Millipore, Billerica, MA, USA) following the manufacturer’s protocol. Briefly, HBMECs were lysed in complete RIP lysis buffer, after which 100 μl of whole cell extract was incubated with RIP buffer containing magnetic beads conjugated with human anti-Ago2 antibody or control IgG. Samples were incubated with Proteinase K (1X), and then immunoprecipitated RNA was isolated. The RNA concentration was measured using a NanoDrop (Thermo Scientific). Moreover, purified RNA was subjected to qRT-PCR analysis to demonstrate the presence of the binding targets using respective primers.

### Biotin pull-down assay

HBMECs were transfected with biotinylated wild-type (Wt) miR-1207-5p, mutant miR-1207-5p (Genomeditech, Shanghai, China). First, cell lysates were harvested 48 h after transfection and incubated with Dynabeads M-280 Streptavidin (Invitrogen, CA, USA) for 3 h at 4 °C according to the manufacturer’s protocol. Then, the beads were washed three times with ice-cold lysis buffer and once with high-salt buffer according to a previous method [18]. The bound RNAs were purified using an RNA extraction kit (Solarbio, Beijing, China) for the qRT-PCR analysis.

### Mouse brain metastasis models

All experimental protocols were approved by the Laboratory Animal Ethical Committee at Chengdu Medical College. Female nude mice (5 weeks old, purchased from Chengdu Dossy Experimental Animals Co, Ltd, Sichuan, China) were injected intracardially with control A549, lnc-MMP2-2-knocked down A549, TGF-β1-pretreated (10 ng/ml for 24 h) control A549, or TGF-β1-pretreated lnc-MMP2-2-knocked down A549 cells (1 × 10^6^ cells in 100 μl PBS) and were monitored for brain metastasis using an IVIS Imaging System. Approximately 3 weeks post-injection, all the mice were euthanized, and whole brains were collected. The presence of brain metastases was confirmed by fluorescence photography and hematoxylin-eosin staining.

### Statistical analysis

Each in vitro experiment was performed independently at least three times. The results are presented as means ± SD. Student’s *t* tests was used for the comparison of two groups. One-way analysis of variance was conducted for comparison of multiple groups. All statistical analyses were performed with GraphPad Prism 5 (GraphPad Software, San Diego, CA, USA). Statistical significance was assigned at *p* < 0.05 (*), *p* < 0.01 (**), or *p* < 0.001 (***).

## Results

### TGF-β1-mediated A549-derived exosomes promote endothelial-to-mesenchymal transition (EndoMT), downregulate tight junction protein expression, and increase HBMECs monolayer permeability

**Fig. 1:**
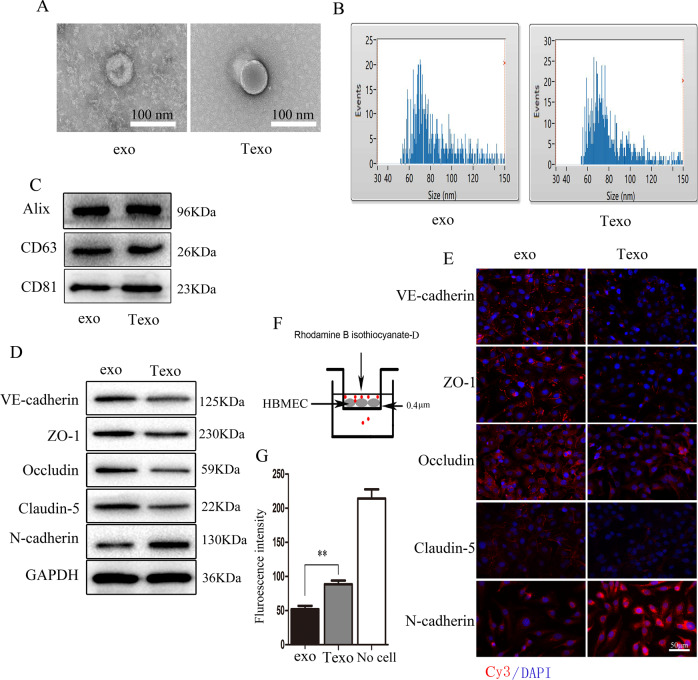
TGF-β1-mediated A549-derived exosomes promote EndoMT, downregulate the expression of tight junction proteins, and increase HBMECs monolayer permeability. **A** TEM images of exosomes secreted by A549 cells (exo) and of TGF-β1-pretreated (10 ng/ml, 24 h) A549 cells (Texo). **B** The size distribution and concentration of exosomes are assessed by nanoparticle tracking analysis (NTA). **C** The expression of exosomes characteristic proteins Alix, CD81, and CD63 are detected using western blotting. **D**, **E** Expression of EndoMT markers (VE-cadherin, N-cadherin) and tight junction proteins (ZO-1, occludin, and claudin-5) in exo- and Texo-treated HBMECs as measured using IF (scale bar, 50 μm) and western blotting assays. **F** Schematic representation of the Transwell chamber used for assaying transport across an endothelial monolayer. **G** The permeability of HBMECs (exo or Texo pre-cocultured) monolayer grown on 0.4-μm filters as measured according to the appearance of rhodamine B isothiocyanate-dextran, which was added in the upper well at the beginning of the experiment and in the bottom well after 2-h incubation. **p < 0.01.

Our previous studies have shown that TGF-β1-mediated A549 cell exosomes may increase lung vascular EC permeability and downregulate its tight junctions [12]. Figure 1A shows TEM images of exosomes first extracted from untreated A549 cell culture media (exo) and TGF-β1-pretreated A549 cell culture media (Texo). Their size distribution was determined using nanoparticle tracking analysis is shown in Fig. 1B. These data showed that no obvious difference exists between the two exosomes in appearance and size. Additionally, the exosomes characteristic proteins Alix, CD9, and CD63 detected using western blotting are shown in Fig. 1C. This analysis revealed that the size, concentration, and characteristic protein expression between exo and Texo were similar. IF staining and western blot analysis revealed that Texo, but not exo, downregulated expression of the endothelial marker VE-cadherin and upregulated expression of the mesenchymal marker N-cadherin. A decreased expression of tight junction proteins (ZO-1, occludin, and claudin-5) was also observed in HBMECs (Fig. 1D, E). We then performed an in vitro permeability assay by measuring the traversing of rhodamine-labeled dextran through HBMECs monolayers growing on 0.4 μm filters. The results showed that vascular EC permeability was markedly higher in Texo than that in exo (Fig. 1F, G).

### TGF-β1-mediated A549-derived exosomes alter the integrity of BBB in vivo

**Fig. 2:**
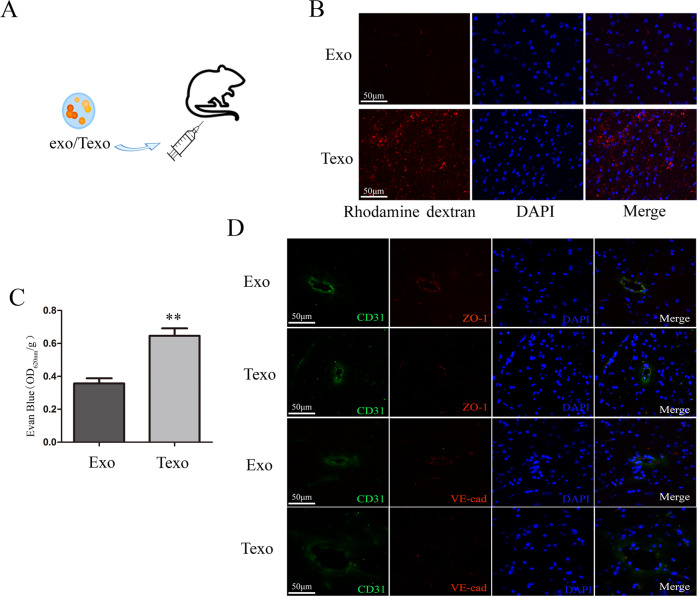
TGF-β1-mediated A549-derived exosomes alters the integrity of the BBB in vivo. **A** Exo or Texo is used for tail vein injection per mouse. **B**, **C** Rhodamine B and Evan blue were injected into the mouse tail vein 24 h after exosomes injection. After 3 h of circulation, **B** in vivo cerebral vascular permeability is determined according to the appearance of intravenously injected rhodamine-dextran (red; scale bar, 50 μm). **C** The absorbance of the Evan blue leakage is detected as OD_620 nm_/g. **D** Animals are euthanized, and tissues are harvested 27 h after exosomes injection. Brain tissues are subjected to double-label IF for analysis of CD31 (green) and ZO-1 (red) or CD31 (green) and VE-cadherin (red) expression (scale bar, 50 μm). **p < 0.01.

Next, to further demonstrate the effect of Texo in in vivo BBB, we injected Texo or exo into the tail vein of mice and examined the integrity of BBB after exosomes treatment (Fig. 2A). The in vivo integrity of BBB was determined according to the appearance of intravenously injected rhodamine-dextran and EB and the expression of tight junction proteins. Notably, Texo significantly enhanced the brain vascular permeability (Fig. 2B, C) and diminished ZO-1 and VE-cadherin expression in CD31^+^ ECs (Fig. 2D).

### lnc-MMP2-2 inhibition in Texo reverses Texo-induced downregulation of tight junction proteins and HBMECs monolayer permeability

A previous study showed that exosomes play important roles as carriers of intercellular signals during vascular remodeling and cancer invasion [19]. Given the role of exosomal lnc-MMP2-2 in regulating HMVEC-L monolayer permeability [12], we explored its role in HBMECs. Exosomes in Texo were isolated, and lnc-MMP2-2 Smart Silencer were loaded in exosomes using Exo-Fect Exosome Transfection Kit. As expected, Texo, which was transfected with lnc-MMP2-2 Silencer, upregulated the expression of tight junction proteins in vitro and in vivo and attenuated the permeability of HBMECs monolayer (Fig. S1).

### lnc-MMP2-2 promotes EndoMT, destroys tight junctions, and induces HBMECs monolayer permeability in vitro

**Fig. 3:**
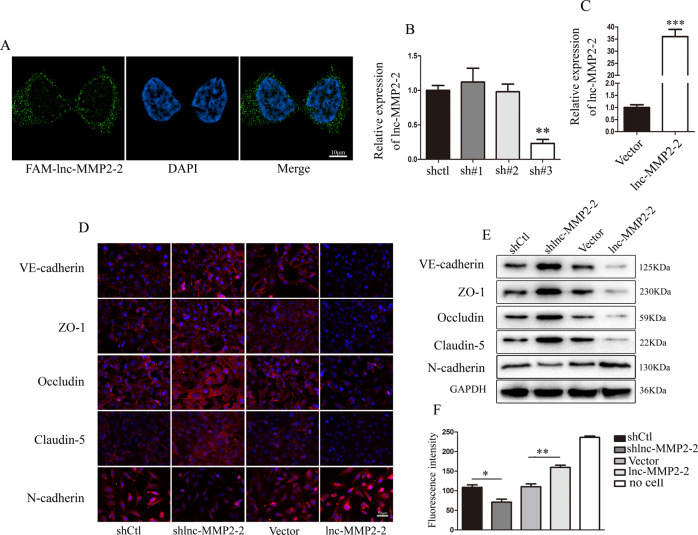
lnc-MMP2-2 promotes EndoMT, destroys tight junctions, and induces HBMECs monolayer permeability in vitro. **A** FISH analysis of lnc-MMP2-2 location in HBMECs. **B**, **C** Infection efficiency of lnc-MMP2-2 silencing and overexpression lentivirus in HBMECs detected using qRT-PCR. **D**, **E** Expression of EndoMT markers (VE-cadherin, N-cadherin) and of tight junction proteins (ZO-1, occludin, and claudin-5) in lnc-MMP2-2-silenced or -overexpressed HBMECs as measured using IF (scale bar, 50 μm) and western blotting assays. **F** HBMECs monolayer permeability in stably infected cells with lnc-MMP2-2 knockdown or overexpression. *p < 0.05, **p < 0.01, ***p < 0.001.

To directly elucidate the biological roles of lnc-MMP2-2 in BBB, we first examined the distribution of lnc-MMP2-2 in HBMECs. The FISH assay showed that lnc-MMP2-2 was mainly expressed in the cytoplasm (Fig. 3A). Next, HBMECs were infected with lnc-MMP2-2 overexpression and silencing lentivirus. Stably infected cells were selected using puromycin, and lnc-MMP2-2 expression was confirmed using qRT-PCR (Fig. 3B, C). Based on these data, shlnc-MMP2-2 #3 was selected for all subsequent experiments. IF staining and western blotting showed that lnc-MMP2-2 overexpression markedly downregulated the levels of the endothelial marker VE-cadherin but upregulated those of mesenchymal markers (N-cadherin and the tight junction proteins ZO-1, claudin-5, and occludin; Fig. 3D, E). Moreover, permeability assays revealed that lnc-MMP2-2 overexpression significantly increased HBMECs monolayer permeability (Fig. 3F). Interestingly, lnc-MMP2-2 silencing showed the opposite effects (Fig. 3D–F).

### lnc-MMP2-2 functions as a molecular sponge for miR-1207-5p in HBMECs

**Fig. 4:**
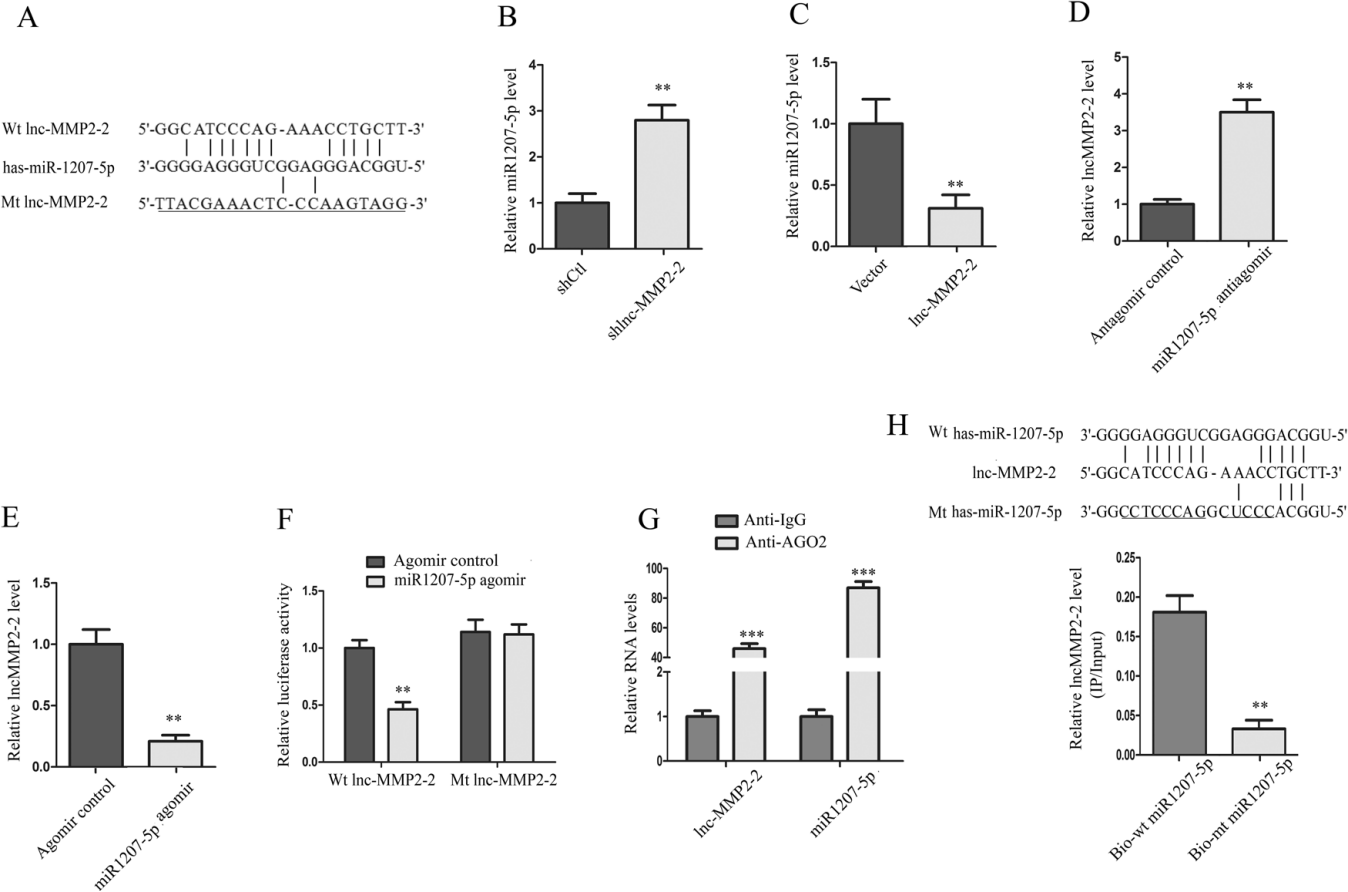
lnc-MMP2-2 functions as molecular sponge for miR-1207-5p in HBMECs. **A** The potential binding sites between miR-1207-5p and lnc-MMP2-2. **B**, **C** Stable lnc-MMP2-2-knocked down or -overexpressed HBMEC are subjected to qRT-PCR for analysis of miR-1207-5p expression. **D**, **E** lnc-MMP2-2 expression is decreased after transfection with miR-1207-5p inhibitor or agomir. **F** Complementary sequence between miR-1207-5p and wild-type (Wt) lnc-MMP2-2. The putative binding sites of miR-1207-5p is mutated in lnc-MMP2-2 (Mt lnc-MMP2-2). Luciferase activity is measured in HBMECs that are co-transfected with miR-1207-5p agomir and Wt or Mt lnc-MMP2-2 vector. **G** Anti-Ago2 RIP is performed in HBMECs transiently overexpressing miR-1207-5p. **H** The sequences for Wt and Mt forms of miR-1207-5p are shown. lnc-MMP2-2 is highly enriched in the sample pulled down by biotinylated Wt miR-1207-5p but not in Mt miR-1207-5p. **p < 0.01, ***p < 0.001.

To identify the potential mechanisms by which lnc-MMP2-2 functioned in HBMECs, bioinformatic tools (microRNA.org and miRBase) were employed to analyze the potential targets of lnc-MMP2-2. The results revealed that miR-1207-5p had putative lnc-MMP2-2 binding sites (Fig. 4A). The qPCR assay showed that knockdown of lnc-MMP2-2 significantly increased miR-1207-5p expression, whereas ectopic lnc-MMP2-2 markedly inhibited miR-1207-5p expression (Fig. 4B, C). Interestingly, knockdown or overexpression of miR-1207-5p also affected lnc-MMP2-2 expression (Fig. 4D, E). Moreover, luciferase reporter assay revealed that miR-1207-5p overexpression lowered the luciferase activity of pGL3-lnc-MMP2-2-Wt but not that of pGL3-lnc-MMP2-2-mut (Fig. 4F).

In addition, the anti-Ago2 RIP assay further confirmed that miR-1207-5p was a target of lnc-MMP2-2 in HBMECs (Fig. 4G). Subsequently, biotin-labeled pull-down assays showed markedly higher lnc-MMP2-2 expression in HBMECs transfected with biotin-labeled miR-1207-5p than that in those labeled with the mutagenesis of the binding sites for lnc-MMP2-2 in miR-1207-5p (Fig. 4H). Collectively, these data demonstrated that lnc-MMP2-2 could directly bind to miR-1207-5p in HBMECs and showed a reciprocal repression of miR-1207-5p and lnc-MMP2-2.

### MiR-1207-5p upregulates the expression of tight junction proteins and attenuates HBMECs monolayer permeability

Next, we confirmed the roles of miR-1207-5p in regulating HBMECs monolayer permeability. miR-1207-5p silencing and knockdown following transfection was confirmed via qRT-PCR (Fig. S2A). IF staining and western blotting showed that miR-1207-5p inhibition markedly lowered the levels of the endothelial marker VE-cadherin but upregulated those of the mesenchymal marker N-cadherin. It also downregulated the expression of the tight junction proteins ZO-1, claudin-5, and occludin (Fig. S2B, C). Moreover, permeability assays revealed that miR-1207-5p inhibition significantly increased HBMECs monolayer permeability (Fig. S2D). As expected, miR-1207-5p overexpression showed the opposite effect (Fig. S2B–D).

### EPB41L5 is a direct target of miR-1207-5p in HBMECs

**Fig. 5:**
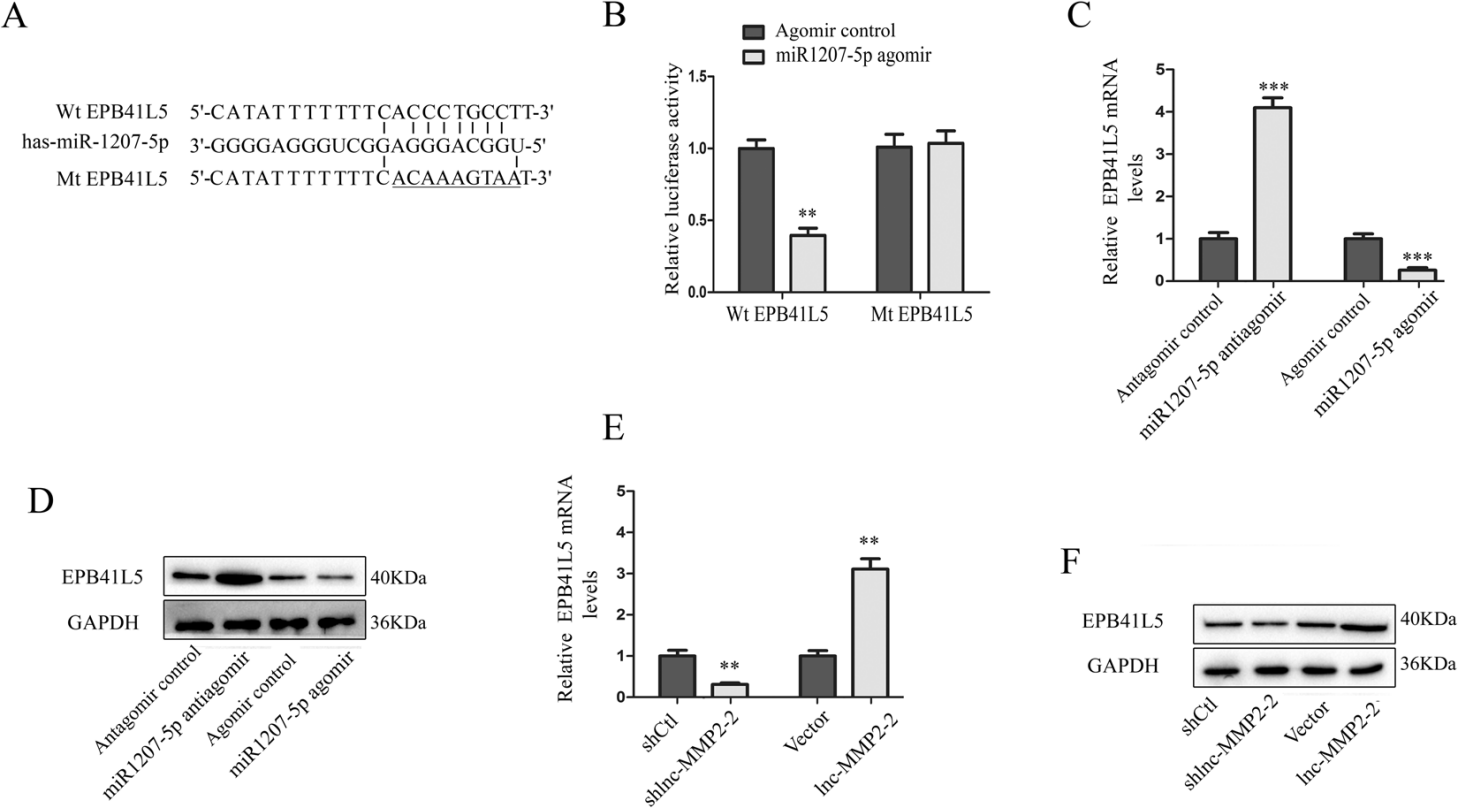
EPB41L5 is a direct target of miR-1207-5p in HBMECs. **A** Bioinformatics analysis showed that miR-1207-5p could directly target the 3′-UTR of EPB41L5 wild-type (Wt). EPB41L5-mutant (Mt) means mutation of binding sites in the 3′-UTR of EPB41L5. **B** miR-1207-5p negatively regulates the luciferase activity of Wt EPB41L5, rather than that of Mt EPB41L5 in HBMECs. **C**, **D** miR-1207-5p inversely regulates the level of EPB41L5 mRNA and protein in HBMECs. **E**, **F** lnc-MMP2-2 positively regulates the abundance of EPB41L5 mRNA and protein in HBMECs. **p < 0.01, ***p < 0.001.

To elucidate the underlying molecular mechanism by which miR-1207-5p exerts its effects on HBMECs, bioinformatic tools (microRNA.org and miRBase) were used for searching the candidate targets of miR-1207-5p. As shown in Fig. 5A, binding sequences of miR-1207-5p were identified in the 3′ untranslated region of EPB41L5 mRNA. Subsequent luciferase reporter assays, qRT-PCR, and western blot analysis revealed that miR-1207-5p could directly target EPB41L5 and negatively modulate the expression of EPB41L5 in HBMECs (Fig. 5B–D). In addition, we explored whether lnc-MMP2-2 could regulate the expression of EPB41L5. As shown in Fig. 5E, F, EPB41L5 expression could be positively regulated by lnc-MMP2-2 in HBMECs. This suggests that EPB41L5 is a direct target of miR-1207-5p and is positively modulated by lnc-MMP2-2 in HBMECs.

### EPB41L5 promotes EndoMT, destroys tight junctions, and induces HBMECs monolayer permeability

**Fig. 6:**
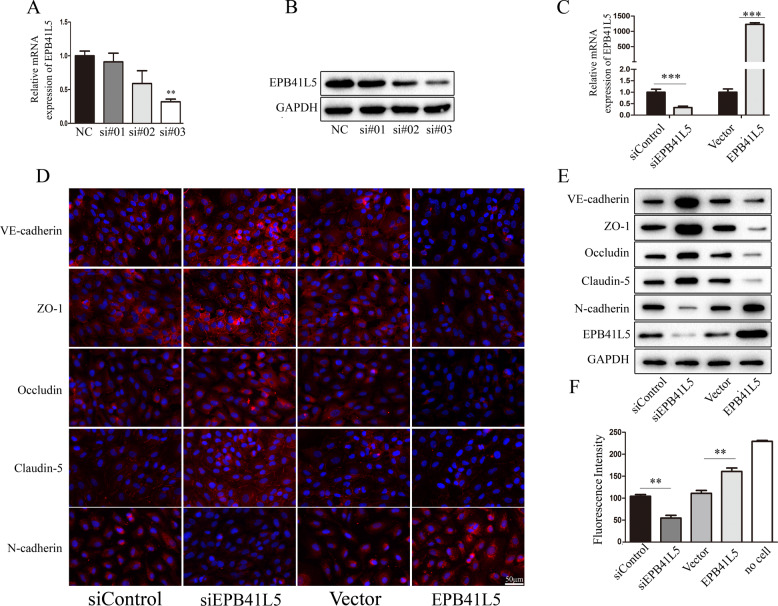
EPB41L5 promotes EndoMT, destroys tight junctions, and induces HBMECs monolayer permeability. **A**, **B** The expression of EPB41L5 in HBMECs transfected with EPB41L5 siRNAs is assessed using qPCR and western blotting. **C** The expression of EPB41L5 in HBMECs effectively transfected with EPB41L5 siRNA (si#3) or EPB41L5 overexpression plasmids is assessed using qPCR. **D**, **E** The expression of EndoMT markers (VE-cadherin, N-cadherin) and of tight junction proteins (ZO-1, occludin, and claudin-5) in EPB41L5 silenced or overexpressed HBMECs as measured by IF (scale bar, 50 μm) and western blotting assays. **F** The permeability of HBMECs monolayers with EPB41L5 knockdown or overexpression. **p < 0.01, ***p < 0.001.

EPB41L5 has been confirmed to play an oncogenic role in glioblastoma [20], gastric cancer [21], and breast cancers [22]. To explore whether EPB41L5 is also critical for BBB permeability, EPB41L5 overexpression or knockdown assays were performed in HBMECs. First, we identified an effective RNAi oligonucleotide to silence EPB41L5 expression in HBMECs (Fig. 6A, B). EPB41L5 knockdown and overexpression were confirmed via qRT-PCR (Fig. 6C). Subsequent IF staining, western blotting, and monolayer permeability analysis revealed that EPB41L5 overexpression significantly promotes EndoMT, destroys tight junctions, and induces HBMECs monolayer permeability (Fig. 6D–F).

### lnc-MMP2-2 knockdown suppresses NSCLC brain metastasis in vivo

**Fig. 7:**
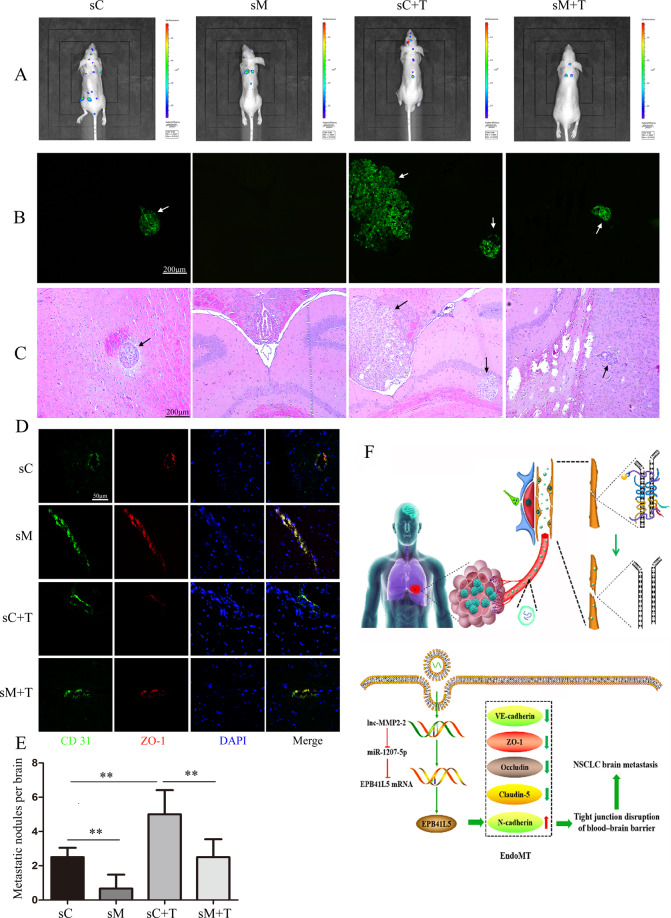
lnc-MMP2-2 knockdown inhibits the NSCLC brain metastasis in vivo. A549 cells stably transfected with control and shlnc-MMP2-2 lentivirus and treated with or without 10 ng/ml TGF-β1 for 24 h. All the cells are then injected into the heart of nude mice (*N* = 6). Three weeks after the injection, mice are photographed and sacrificed. **A** Representative IVIS imaging in brain metastasis mice. **B**, **C** Representative bioluminescent images of brain metastases and paired HE images (scale bar, 200 μm). **D** Brain tissues are subjected to double-label IF for analysis of ZO-1 (red) expression in CD31^+^ (green) endothelial cells (scale bar, 50 μm). **E** The average number of metastatic nodules in the brain metastasis mice. **p < 0.01. **F** Schematic of the proposed mechanism of action of the lnc-MMP2-2 /miRNA-1207-5p/EPB41L5 axis. A detailed description is shown in the main text. sC: shControl, sM: shlnc-MMP2-2, sC + T: shControl + TGF-β1, sM + T: shlnc-MMP2-2 + TGF-β1.

To further elucidate the effect of lnc-MMP2-2 in NSCLC brain metastasis in vivo, a brain metastasis mouse model was established by implanting stably transfected A549 cells with control or shlnc-MMP2-2 lentivirus treated with or without TGF-β1. lnc-MMP2-2 knockdown significantly inhibited the occurrence of brain metastasis (Fig. 7A–E).

## Discussion

BBB permeability is highly increased during lung cancer brain metastasis, but the mechanism still remains largely unknown. The current study found that TGF-β1-mediated NSCLC-derived exosomes promote EndoMT, downregulate the expression of tight junction proteins, increase HBMECs monolayer permeability, and alter the integrity of BBB in vivo. Inhibition of exosomal lnc-MMP2-2 reversed this effect. lnc-MMP2-2 overexpression and knockdown assays showed that lnc-MMP2-2 remarkably promotes EndoMT, destroys tight junctions, and induces HBMECs monolayer permeability.

Despite recent advances in treatment modalities, lung cancer remains the leading cause of cancer-related deaths worldwide, and metastasis remains the major determinant of poor outcomes [23]. A previous study showed that brain metastasis eventually occurs in ~40% of NSCLC patients, and 10% of NSCLC patients have brain metastasis at the time of diagnosis. The BBB is a barrier between blood circulation and brain tissue that provides anatomical and physiological protection for the CNS, supplies brain tissue with nutrients, and restricts the flow of essential components in and out of the CNS [24]. The BBB permeability is highly increased in lung cancer brain metastases [5], enabling the penetration of circulating tumor cells into the brain and promoting brain metastases. However, the mechanisms by which the BBB permeability is altered remain incompletely understood.

Human brain microvascular ECs is the main component of BBB. Thus, the cell morphology and tight junctions between HBMECs is essential for BBB permeability. EndoMT is a specific form of epithelial-to-mesenchymal transition characterized by a loss of endothelial features and the acquisition of mesenchymal features. Studies have shown that TGF-β1 is a potent inducer of EndoMT [25, 26]. TGF-β1-mediated lncRNAs have been shown to play important regulatory roles in cancer progression [27–29]. Interestingly, our previous study showed that TGF-β1-mediated exosomal lnc-MMP2-2 promotes lung cancer cell invasion into the vasculature by regulating the permeability of lung vascular ECs [12]. Thus, we hypothesized that TGF-β1-mediated exosomal lnc-MMP2-2 might be a regulator in HBMECs and eventually induce BBB dysfunction.

One of the most well-known function of lncRNAs is that they function as competing endogenous RNAs (ceRNAs) to sponge microRNAs [30, 31]. The location of lncRNA is closely related to its function. For example, nuclear localization is related to its transcriptional modulator effect, and cytoplasmic localization can play the role of ceRNA [31]. We particularly detected lnc-MMP2-2 location in HBMECs and found that lncMMP-2-2 is mainly expressed in the cytoplasm; this result corresponds to its ceRNA mechanism. Further, our bioinformatic analyses revealed that miR-1207-5p had putative lnc-MMP2-2 binding sites. lnc-MMP2-2 knockdown markedly upregulated miR-1207-5p expression, whereas lnc-MMP2-2 overexpression downregulated miR-1207-5p expression. Moreover, luciferase reporter assay, biotin pull-down assay, and RIP revealed that miR-1207-5p was a target of lnc-MMP2-2 in HBMECs. Thus, we considered that lnc-MMP2-2 increases the permeability of vascular ECs in the BBB by downregulating miR-1207-5p expression.

EPB41L5 belongs to the NBL4 subgroup of the band 4.1 superfamily, which has a conserved 4.1-ezrin-radixin-moesin (FERM) domain at the N-terminus and a nonhomologous sequence at the C-terminus [20]. Recent studies confirmed that EPB41L5 is involved in the occurrence and development of squamous cell carcinoma [32], gastric cancer [21], and breast cancer [22]. In this study, EPB41L5 was identified as a direct target of miR-1207-5p in HBMECs. Notably, lnc-MMP2-2 positively regulated EPB41L5 expression in HBMECs, while miR-1207-5p showed an opposite regulatory effect. Importantly, both ectopic EPB41L5 or MiR-1207-5p silencing in HBMECs directly promoted EndoMT, destroyed tight junctions, and induced HBMECs monolayer permeability.

Furthermore, this study has some limitations. Endothelial glycocalyx, a carbohydrate-rich layer that lines the luminal side of the vascular endothelium, is essential in vascular permeability and is regarded as the first BBB barrier. [33]. Numerous experiments have proved that the glycocalyx abscission is related to the pathophysiology of inflammatory diseases, atherosclerosis, and cancer metastases [34–36]. However, few studies have shown the effect of long non-coding RNA in regulating endothelial glycocalyx. Therefore, we only focused on the vascular EC without involving endothelial glycocalyx in this research. Hence, the role of lnc-MMP2-2 in regulating endothelial glycocalyx remains unclear, which is our future study plan.

In conclusion, we identified a novel ceRNA regulatory pathway in which lnc-MMP2-2 upregulates EPB41L5 expression by sponging miR-1207-5p. EPB41L5 directly promotes EndoMT, destroys tight junctions, increases BBB permeability, ultimately promoting brain metastasis in NSCLC (Fig. 7F). These findings support the potential of exosomal lnc-MMP2-2 as a novel biomarker and therapeutic target against NSCLC brain metastasis.

## Supplementary information

Supplemental Figure legends

Supplemental Figure 1

Supplemental Figure 2
