# Correction: TGF-β1-mediated exosomal lnc-MMP2-2 increases blood–brain barrier permeability via the miRNA-1207-5p/EPB41L5 axis to promote non-small cell lung cancer brain metastasis

**DOI:** 10.1038/s41419-021-04072-1

**Published:** 2021-10-07

**Authors:** Dongming Wu, Shihua Deng, Li Li, Teng Liu, Ting Zhang, Jing Li, Ye Yu, Ying Xu

**Affiliations:** 1 School of Clinical Medicine, Chengdu Medical College, Chengdu, Sichuan PR China; 2 The First Affiliated Hospital of Chengdu Medical College, Chengdu, Sichuan PR China

Correction to: *Cell Death and Disease* 10.1038/s41419-021-04004-z, published online 20 July 2021

The original version of this article unfortunately contained a mistake. Due to a typesetting error the following sentence was omitted: “These authors contributed equally: Dongming Wu, Shihua Deng”. We apologize for the error. The original article has been corrected.

