# Supplementary material for: TGF-β1-mediated exosomal lnc-MMP2-2 increases blood–brain barrier permeability via the miRNA-1207-5p/EPB41L5 axis to promote non-small cell lung cancer brain metastasis
Source: Cell Death Dis. 2021 Jul 20;12(8):721. doi: 10.1038/s41419-021-04004-z (PMC8292445; doi:10.1038/s41419-021-04004-z)
Supplement: Supplementary file 1 — Supplemental Figure legends [file 41419_2021_4004_MOESM1_ESM.doc]

**Supplemental Figure legends**

**Figure S1.** lnc-MMP2-2 inhibition in Texo reverses Texo-induced tight-junction destruction in vivo and HBMECs monolayer permeability. A. Control Texo (Texo+NC) or lnc-MMP2-2-silenced Texo (Texo+silnc-MMP2-2) are used for tail-vein injection per mouse. Animals are euthanized, and brain tissues are harvested 24 h after exosome injection. Brain tissues are then subjected to double-label IF for CD31 (green) and ZO-1 (red) analysis. B−C. Expression of EndoMT markers (VE-cadherin, N-cadherin) and tight junction proteins (ZO-1, occludin, claudin-5) in Texo+NC-or Texo+silnc-MMP2-2-treated HBMECs as measured using IF and western blotting assays. D. The permeability of Texo+NC- or Texo+silnc-MMP2-2-treated HBMECs monolayer.

**Figure S2.** miR-1207-5p upregulates the expression of tight junction proteins and attenuates HBMECs monolayer permeability. A. The expression of miR-1207-5p in HBMECs transfected with miR-1207-5p inhibitor or agomir as assessed using qPCR. B-C. The expression of EndoMT markers (VE-cadherin, N-cadherin) and tight junction proteins (ZO-1, occludin, and claudin-5) in HBMECs transfected with miR-1207-5p inhibitor or agomir as measured using IF (scale bar, 50 μm) and western blotting assays. D. The permeability of HBMECs monolayer transfected with miR-1207-5p inhibitor or agomir.
