# Supplementary figures and images for: TGF-β1-mediated exosomal lnc-MMP2-2 increases blood–brain barrier permeability via the miRNA-1207-5p/EPB41L5 axis to promote non-small cell lung cancer brain metastasis
Source: Cell Death Dis. 2021 Jul 20;12(8):721. doi: 10.1038/s41419-021-04004-z (PMC8292445; doi:10.1038/s41419-021-04004-z)

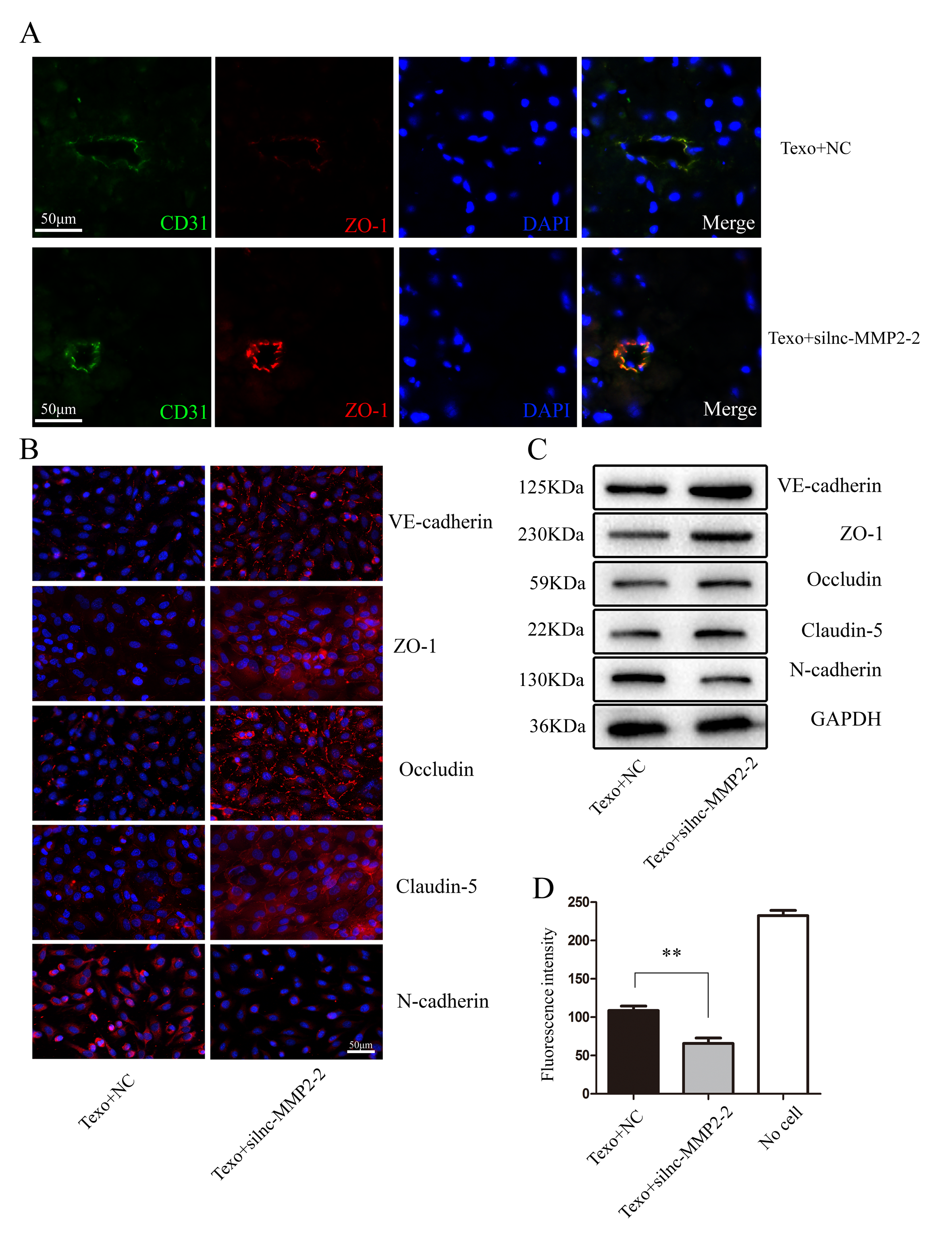

Supplement: Supplementary file 2 — Supplemental Figure 1 [file 41419_2021_4004_MOESM2_ESM.tif]

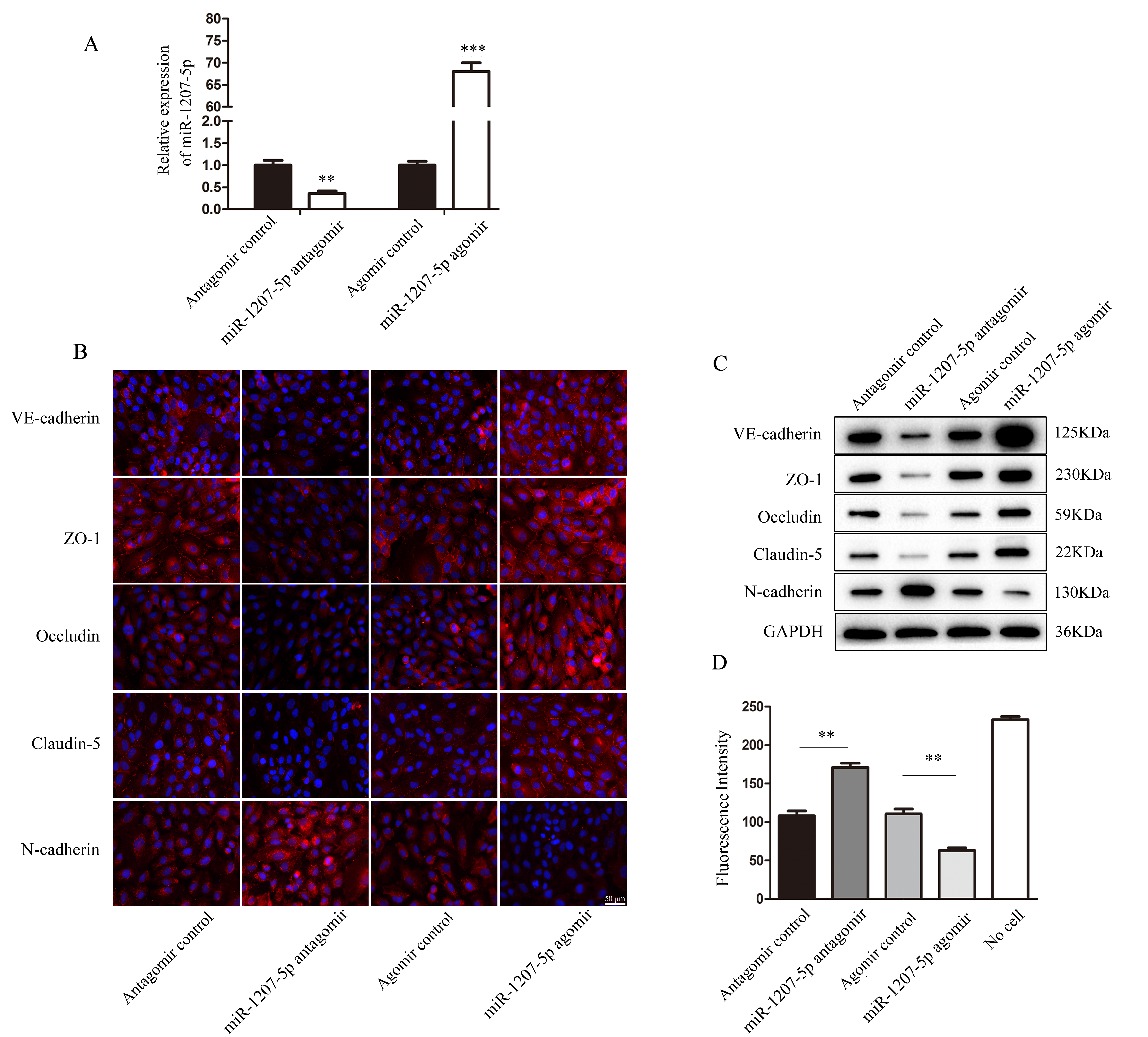

Supplement: Supplementary file 3 — Supplemental Figure 2 [file 41419_2021_4004_MOESM3_ESM.tif]
